# siRNA against the G gene of human metapneumovirus

**DOI:** 10.1186/1743-422X-9-105

**Published:** 2012-07-10

**Authors:** Faith Maxine Preston, Claire P Straub, Ruben Ramirez, Suresh Mahalingam, Kirsten M Spann

**Affiliations:** 1 Clinical Medical Virology Centre, The University of Queensland, Brisbane, QLD 4072, Australia; 2 Sir Albert Sakzewski Virus Research Centre, Children’s Health Services, Brisbane, QLD 4029, Australia; 3 Faculty of Applied Science, University of Canberra, Canberra, ACT 0200, Australia; 4 Institute for Glycomics, Griffith University, Gold Coast, QLD 4222, Australia

**Keywords:** siRNA, hMPV, Type I interferon

## Abstract

**Background:**

Human metapneumovirus (hMPV) is a significant viral respiratory pathogen of infants and children, the elderly and immunocompromised individuals. Disease associated with hMPV infection resembles that of human respiratory syncytial virus (RSV) and includes bronchiolitis and pneumonia. The glycosylated G attachment protein of hMPV is required for viral entry *in vivo* and has also been identified as an inhibitor of innate immune responses.

**Findings:**

We designed and validated two siRNA molecules against the G gene using A549 cells and demonstrated consistent 88-92% knock-down for one siRNA molecule, which was used in subsequent experiments. Significant reduction of G mRNA in A549 cells infected with hMPV did not result in a reduction in viral growth, nor did it significantly increase the production of type I interferon (α/β) in response to infection. However, there was a moderate increase in IFN-β mRNA expression in response to infection in siG-transfected cells compared to untransfected and si-mismatch-transfected cells. Expression of G by recombinant adenovirus did not affect type I IFN expression.

**Conclusion:**

G has been previously described as a type I interferon antagonist, although our findings suggest it may not be a significant antagonist.

## Findings

### Background

Human metapneumovirus (hMPV) is a member of the *Pneumoviridae* subfamily of the *Paramyxoviridae*[1]. hMPV infects half of all infants under the age of 1 year and almost all children have been infected by the age of 10 years [2,3]. hMPV causes acute respiratory disease in children worldwide [4-6]. Currently, there are no vaccines or targeted therapies for hMPV infection. RNA interference (RNAi) is a mechanism of post-transcriptional gene silencing found in almost all eukaryotes and is triggered by endogenous small non-coding microRNAs (miRNAs) or small interfering RNAs (siRNAs) [7]. The recently development of synthetic sequence-specific siRNAs has allowed a variety of host and pathogen genes to be targeted for mRNA cleavage, effectively silencing or significantly reducing gene expression [7]. Reports for RSV and hMPV suggest that RNAi can inhibit viral mRNA expression, and is a potentially effective therapy for respiratory infections [8-13].

siRNA molecules targeting the nucleoprotein (N) and phosphoprotein (P) mRNA of hMPV have been found effective at inhibiting the hMPV genome [13]. Here we have designed and validated a siRNA molecule against the G gene, which encodes a principal attachment protein required for replication *in vivo* and also identified as a type I interferon (IFN) antagonist [14].

### Design and validation of siRNA against the G gene

The hMPV isolate CAN97-83 G gene was used as target sequence to design siRNA molecules by Dharmacon, using their algorithm and ON-TARGETplus ® sense strand modification to avoid off-target effects. Two siRNA molecules were chosen for analysis and validation:

G1: sense - GCUCAAAGCAAGAGUGAAA

G2: sense - AGGUGAAAGUAGAGAACAUUU

The molecules do not target all isolates of hMPV as identified by BLAST, due to the high level of divergence in the G sequence [6,15]. A mis-matched siRNA molecule (siMM; sense - CUAAAGUGGUAGUUGAUAUUU) was also generated as a control for the effect of transfection and off-target effects. hMPV (CAN97-83) was propagated in LLC-MK2 cells, concentrated through a 30%/60% w/v sucrose gradient, pelleted by centrifugation at 12,000 x *g* for 2 hours at 4 °C, and the viral titre quantified using a TCID_50_ assay and anti-hMPV M monoclonal antibody (Chemicon) to identify positive cells. Preliminary transfection experiments (not shown) using a fluorescein labeled RNAi delivery control (Mirus) demonstrated that transfection of 200 mM siRNA resulted in 100% transfection efficiency at 24 h. Preliminary infection experiments established that 100% of cells were infected by 24 h post-infection (pi) when exposed to hMPV at a multiplicity of infection (MOI) of 2 in the absence of trypsin.

A549 cells were transfected with siG1, siG2 or siMM 6 h prior to infection with hMPV. At 12 h and 24 h pi, total RNA was extracted from cells using TRIzol/chloroform separation and RNeasy spin columns (QIAGEN). cDNA was generated using superscript III reverse transcriptase (Invitrogen) and oligo d(T)_20_ primers to select for mRNA. Quantitative (q) RT-PCR was performed to quantify G mRNA reduction using the following oligonucleotides: forward primer 3′- GCAGCAATAGACATGCTCAAA-5′, reverse primer 3′-GAGCTGGTGTGGTGTTCTGA-5′, hybridization probe 3′-HEX-AAATCGTGTGGCACGTAGCAAATGC-BHQ-5′. N mRNA was also quantified using the following oligonucleotides: forward primer 3′- TTACGGTGCTGGTCAAACAA-5′, reverse primer 3′-TTTGGGCTTTGCCTTAAATG-5′, hybridization probe 3′-HEX-CTATGACCTGGTGCGAGAAATGGGC-BHQ-5′. qPCR was performed using QuantiTECT mastermix (QIAGEN) and a Rotorgene 3000 (Roche). G and N mRNA in hMPV-infected cells was quantified in relation to β-actin mRNA for each sample and in relation to untransfected cells using the ΔΔCt formula. The ΔΔCt value was then converted to percent reduction of G or N mRNA (Applied Biosystems Technotes vol 15[2] p. 10).

**Figure 1:**
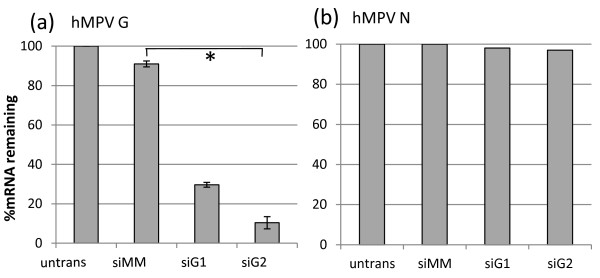
**The effect of prior siG1 and siG2 transfection of A549 cells on G and N mRNA 24 h post-infection with hMPV at a MOI of 2.** (**a**) hMPV G mRNA was significantly reduced by siG2 compared to cells either transfected with siMM (*P = 1.6 x 10^-5^, t-test) or not transfected. siG1 was not as effective at reducing G mRNA and subsequently was not used for experimentation. (**b**) siG1, siG2 and siMM did not reduce nucleoprotein (N) mRNA. hMPV N and G mRNA were quantified by RT-qPCR. Data was normalised to β-actin and expressed relative to untreated A549 cells.

At 12 h pi, siG1 and siG2 resulted in 83.7% and 85.2% G mRNA reduction respectively compared to siMM (not shown). In three independent experiments, 200 mM siG2 resulted in a mean G mRNA reduction of 88% (12% remaining; *P* = 3.4 x 10^-4^ compared to siMM) at 24 h pi in infected A549 cells (Figure 1a). siG1 was not as effective at reducing G mRNA at 24 h pi (29% remaining), and was therefore not used for subsequent experiments. The effect of siG1 and siG2 on hMPV N was negligible (<3%), indicating that other viral genes were not affected (Figure 1b).

We had planned to investigate the effect of siG2 transfection on G protein expression. An antibody to detect G protein in lysed cell preparations has not been described in the literature, nor is commercially available. Affinity purified rabbit antisera were generated to a G peptide considered suitable for this purpose. However, the sera detected only a ~32 kDa band, which may have been a truncated form of G, and did not detect the non-truncated 90 kDa G protein. Due to the ambiguity of G protein detection we were not able to quantify the effect of G mRNA reduction on G protein expression. However, other studies that involve the validation of siRNA molecules against viral mRNAs demonstrate that a reduction in target mRNA similar to that demonstrated here correlate to a significant reduction in protein [9,10,16].

### Knockdown of G does not modulate viral growth in vitro or the induction of type I interferon

**Figure 2:**
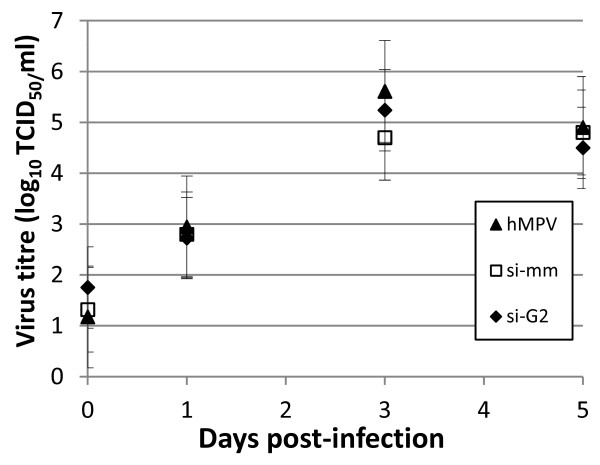
**The effect of siG2 and siMM transfection 6 h prior to infection of A549 cells with hMPV at a MOI of 0.1.** Viral supernatants were collected on the indicated days and the shed hMPV quantified using immunostaining and expressed as TCID_50_/ml. A significant reduction in G mRNA did not affect hMPV shed over 5 days pi.

Triplicate cultures of A549 cells were transfected with siG2, siMM or untransfected, as described above, and infected with hMPV at a MOI of 0.1. Cell supernatants were collected on days 0, 1, 3 and 5 pi, and the titres of shed virus quantified by TCID_50_ assay. Reduction of G mRNA was confirmed at day 1 pi (not shown). There was no significant difference in the titres of shed virus between cells transfected with siMM, siG or untransfected, which indicated that the growth of hMPV *in vitro* was not affected by G knockdown (Figure 2; ANOVA). This correlates to a study using recombinant (r) hMPVΔG in which deletion of G did not attenuate viral growth in LLC-MK2 cells [17]. This differs to a report in which rhMPVΔG displayed a reduction in viral titre by one third to half a log after 24 h of infection in A549 cells [14].

**Figure 3:**
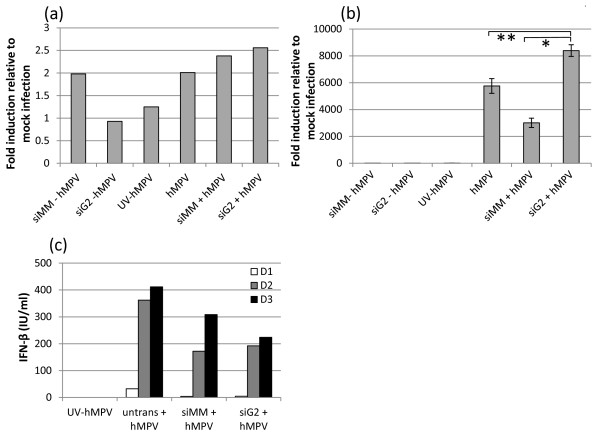
**The effect of G knockdown on type I Interferon induction.** A549 cells were transfected with siMM, siG2 or were not transfected prior to infection with hMPV at a MOI of 2, or mock infection with media. UV-inactivated hMPV was used as a control for interferon induction. Transcription of mRNA encoding human IFN-α (**a**) and IFN-β (**b**) was measured by RT-qPCR 24 h post-infection. There was a moderately significant increase in IFN-β mRNA in siG2 transfected cells compared to untransfected cells (*P = 0.03; t-test) and cells transfected with siMM (**P = 0.02; t-test) prior to hMPV infection. Data was normalised to β-actin and expressed relative to uninfected cells. (**c**) A reduction in G did not affect IFN-β secretion from cells transfected with either siMM or siG2 prior to hMPV infection, compared to untransfected and infected cells. Supernatants were collects on days 1, 2 and 3 post-infection for ELISA analysis.

It has been reported that deletion of G from rhMPV results in an increase in chemokine and type I IFN production in infected airway epithelial cells [14]. Here we investigated if knockdown of G by siRNA would also result in an increase in type I IFN induction. A549 cells were transfected with siMM or siG2, or not transfected and 6 h later infected at a MOI of 2 with hMPV, or mock-infected with media. UV-inactivated hMPV was used as a control for IFN induction. 24 h pi cells were harvested and total RNA extracted as described above. RT-qPCR was performed to quantify IFN-β and -α mRNA using primers and molecular probes previously described [18]. G mRNA knockdown by 88-92% was confirmed by qPCR. The siMM and siG2 molecules alone did not induce either IFN-α or -β indicating no off-target effects on type I IFN induction (Figure 3 a and b, first two bars). hMPV infection alone did not induce IFN-α and did induce IFN-β by 5850-fold over uninfected cells. Transfection with siMM prior to hMPV infection (siMM + hMPV) dampened IFN-β induction indicating some effect of transfection on the cells. However, the knockdown of G resulted in a marginally significant increase in IFN-β mRNA induction compared to hMPV infection alone (*P* = 0.03). A more significant increase was observed compared to siMM + hMPV (*P* = 0.02), although the difference in fold induction between these two treatments, from 3000-fold (siMM) to 8500-fold (siG2), was only 2.85, which may be of only marginal biological significance. ELISA was performed on the cell supernatants collected from transfected and/or infected cells at 24 h, 48 h and 72 h pi. A reduction of G mRNA did not result in a significant increase in secreted IFN-β at any time (Figure 3c; *P* > 0.05). In fact, a slight reduction in secreted IFN-β is observed, which may be the result of transfection. This suggests that although there is a moderate increase in IFN-β mRNA when G mRNA is reduced, this does not result in an increase in IFN-β protein secreted from infected cells. This observation differs from another study in which there was a significant increase in IFN-α/β secretion from infected cells when G was deleted from rhMPV. ELISA (not shown) for IFN-α was conducted and showed no detectable induction as correlated to qPCR results (Figure 3a). Our growth curves for hMPV are similar to those of Biacchesi et al., (2004), which also suggest that a knockdown of G does not induce type I IFN, which may attenuate viral growth.

**Figure 4:**
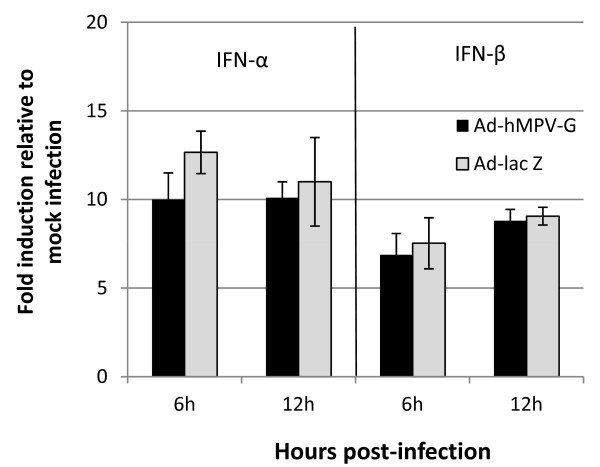
**The effect of Ad-hMPV-G expression on type I IFN induction.** A549 cells were infected with Ad-hMPV-G or Ad-lac z (MOI 1). Cells were collected at the indicated times after infection for the quantification of IFN α/β mRNA transcription by RT-qPCR. hMPV G expression did not affect IFN-α or –β expression at 6 h or 12 h post-infection

The effect of G on type I IFN induction was also investigated using an adenovirus which expressed the hMPV G protein (Ad-hMPV-G; gift from R. Tripp, University of Georgia, Athens, USA). A549 cells were infected with Ad-hMPV G or Ad-Lac Z as a control, at a MOI of 1. After 1 h adsorbtion at 37 °C, virus inoculum was replaced with growth media and infected cells collected for mRNA analysis at 6 h and 12 h pi. G protein expression by adenovirus did not significantly affect type I IFN mRNA levels compared to control (Figure 4).

In summary, we have designed and validate a siRNA molecule that is effective against the G gene of hMPV *in vitro.* A significant reduction in G mRNA did not reduce viral growth *in vitro* or induce a significant type I IFN response, suggesting that G may not be a significant type I IFN antagonist, and other hMPV proteins may play a role in modulating type I IFN induction. However hMPV G may still be a valid target for RNAi as G is required for viral replication *in vivo*[17].

## Competing interests

The authors declare that they have no competing interests.

## Authors’ contributions

KMS and SM conceived of the study. FMP, CPS and RR performed the experiments. FMP and KMS wrote the manuscript. All authors read and approved the final manuscript.
